# Antagonistic Potentiality of Actinomycete-Derived Extract with Anti-Biofilm, Antioxidant, and Cytotoxic Capabilities as a Natural Combating Strategy for Multidrug-Resistant ESKAPE Pathogens

**DOI:** 10.4014/jmb.2211.11026

**Published:** 2022-12-23

**Authors:** Mohamed H. El-Sayed, Fahdah A. Alshammari, Mohammed H. Sharaf

**Affiliations:** 1 Department of Biology, College of Science and Arts, Northern Border University, Saudi Arabia; 2 Department of Botany and Microbiology, Faculty of Science, Al-Azhar University, Cairo 11884, Egypt

**Keywords:** Antibacterial, ESKAPE pathogens, extract, anti-biofilm, confocal microscopy, *Streptomyces lienomycini* BOGE18

## Abstract

The global increase in multidrug-resistant (MDR) bacteria has inspired researchers to develop new strategies to overcome this problem. In this study, 23 morphologically different, soil-isolated actinomycete cultures were screened for their antibacterial ability against MDR isolates of ESKAPE pathogens. Among them, isolate BOGE18 exhibited a broad antibacterial spectrum, so it was selected and identified based on cultural, morphological, physiological, and biochemical characteristics. Chemotaxonomic analysis was also performed together with nucleotide sequencing of the 16S rRNA gene, which showed this strain to have identity with *Streptomyces lienomycini*. The ethyl acetate extract of the cell-free filtrate (CFF) of strain BOGE18 was evaluated for its antibacterial spectrum, and the minimum inhibitory concentration (MIC) ranged from 62.5 to 250 μg/ml. The recorded results from the in vitro anti-biofilm microtiter assay and confocal laser scanning microscopy (CLSM) of sub-MIC concentrations revealed a significant reduction in biofilm formation in a concentration-dependent manner. The extract also displayed significant scavenging activity, reaching 91.61 ± 4.1% and 85.06 ± 3.14% of 2,2-diphenyl-1-picrylhydrazyl (DPPH) and 2,2′-azino-bis(3-ethylbenzothiazoline-6-sulfonic acid) (ABTS), respectively. A promising cytotoxic ability against breast (MCF-7) and hepatocellular (HePG2) cancer cell lines was obtained from the extract with IC_50_ values of 47.15 ± 13.10 and 122.69 ± 9.12 μg/ml, respectively. Moreover, based on gas chromatography-mass spectrometry (GC-MS) analysis, nine known compounds were detected in the BOGE18 extract, suggesting their contribution to the multitude of biological activities recorded in this study. Overall, *Streptomyces lienomycini* BOGE18-derived extract is a good candidate for use in a natural combating strategy to prevent bacterial infection, especially by MDR pathogens.

## Introduction

One of the greatest challenges facing healthcare systems is antibiotic resistance, which has become a serious public health problem. Antibiotic-resistant strains were initially limited to the hospital environment, but now they are widespread. This transformation can be attributed to many factors, including globalization, lack of proper antimicrobial stewardship, overuse of antibiotics in aquaculture and animal husbandry, and use of wide-spectrum antibiotics along with acquisition of antibiotic resistance genes within bacterial populations [1].

Many gram-positive and gram-negative bacterial species that were once considered harmless commensals have now evolved and emerged as serious pathogens that are resistant to the common antibiotics typically used to treat nosocomial and community-acquired infections [2]. Among MDR bacteria are the 'ESKAPE' group, which have been identified as the most notorious pathogens and includes gram-positive (*Enterococcus faecium* and *Staphylococcus aureus*), and gram-negative (*Klebsiella pneumoniae*, *Acinetobacter baumannii*, *Pseudomonas aeruginosa* and *Enterobacter* sp.) species. The WHO has listed ESKAPE species as priority pathogens because they are now the most frequent global cause of skin and soft tissue infections [3].

Many studies have reported that diseases caused by MDR pathogens rank among the world's leading causes of morbidity and mortality [4]. Infections with MDR bacteria, especially ESKAPE pathogens, are extremely difficult to treat and can spread throughout hospital or community environments [5]. As a result, the WHO has established an urgent priority list for new antibiotic discovery [3].

The increasing suffering of patients with infectious diseases caused by pathogenic microorganisms, especially MDR species, prompted the need to discover novel antibiotics by screening microorganisms from natural sources [6]. Microorganisms have made enormous contributions to human health and well-being all around the world. Bacteria can produce many pharmaceutical metabolites, which constitute half of the medications on the market today [7]. Currently, there is growing interest in the health effects exerted by microbial-derived metabolites known as bioactive postbiotic metabolites [8], which can be used for biotechnological applications, specifically in the pharmaceutical industry [9].

Actinomycetes are a popular group of microorganisms that create a wide range of bioactive postbiotic metabolites with diverse activities [10]. Actinomycetes are aerobic, gram-positive bacteria that often have a filamentous and sporulating morphological appearance, and their DNA is composed of more than 55% guanine and cytosine [11]. Approximately 42% of all recorded bioactive compounds of microbial origin have so far been produced from actinomycetes [12], and the majority of these molecules are traceable to soil-dwelling *Streptomyces* [13].

The genus *Streptomyces* has been deeply probed for the discovery of new bioactive metabolites due to its vast genetic and metabolic diversity and its unique ability to catabolize a wide range of substances [14]. In addition to being a key producer of antibiotics, the *Streptomyces* genus has been shown to be a rich source of bioactive-derived metabolites with diverse pharmacological activities, including anti-biofilm [15, 16], antioxidant [17, 18], and anti-cancer compounds [19, 20]. Discovering a *Streptomyces* strain with a multitude of biological activities is therefore of great significance.

Due to the serious infections caused by ESKAPE pathogens, their pattern of antibiotic resistance and the limited reports of antagonistic activity from actinomycetes against them, we sought in this study to isolate and identify an actinomycetes isolate with significant inhibition against MDR-ESKAPE pathogens. Moreover, evaluation of the potential anti-biofilm, antioxidant and cytotoxic capabilities of the extract derived from this isolate was also performed.

## Materials and Methods

### Chemicals and Media

The chemicals used in this study were purchased from Merck (Germany). The culture media, blood agar, brain heart infusion agar (BHIA) and tryptic soy agar/broth (TSA/TSB), were used for isolation and identification of bacterial pathogens, and Mueller-Hinton agar/broth (MHA/MHB) were used for testing antibacterial activity. All were obtained from Oxoid (UK).

### Collection of Clinical Specimens

A total of 415 clinical specimens, including abscess (*n* = 85), throat (*n* = 78), wound (*n* = 90) and tooth (*n* = 62) swabs as well as urine (*n* = 65) and sputum (*n* = 35) cultures, were collected from patients hospitalized in various departments at hospitals in Egypt during the period from December 2021 to April 2022. To avoid drying the collected swabs, they were transported to the microbiology lab within one hour after collection.

### Isolation of Bacterial Pathogens

The collected specimens were immediately inoculated on blood agar plates, incubated at 37°C for 24 h, and then checked for bacterial growth. Plates with no growth were reincubated for an additional 24 h. The grown colonies were picked out and purified by subculturing on BHIA, and the pure isolates were stored at -20°C on slants of TSA media for further investigation.

### Antibiotic Susceptibility Testing (AST) and Identification of MDR Species

A loopful of the pure bacterial culture was suspended in 3.0 ml of sterile saline (aqueous 0.45% NaCl, pH 7.0) using a clear plastic (polystyrene) test tube (12 × 75 mm), and the turbidity was adjusted at 0.5 McFarland standard. AST was carried out with the VITEK 2 Advanced Expert System (AES) [21] and analyses in which antibiotic susceptibility cards were inoculated with the adjusted bacterial suspensions. The VITEK cards AST-Gp67, AST-Gp68, and AST-GN13 were used for staphylococci/enterococci/streptococci, *Streptococcus pneumoniae*, and gram-negative bacteria, respectively. The selected MDR species were identified with the VITEK 2 (bioMérieux, France) system [22] according to the instructions of the manufacturer.

### Soil Sampling and Isolation of Actinomycetes

Fifteen soil samples (approx. 50 g each) were collected from El-Bahariya Oasis [latitude 28.33 49.81°N, longitude 28.46 10.49°E], Western Desert, Giza Governorate, Egypt. The samples were placed in sterile plastic bags, sealed, and then kept in an icebox. The collected soils were pretreated by heating at 70°C for 20 min and mixing with CaCO3 (1:100) for 24 h. Isolation of actinomycetes was carried out using the standard dilution plate method [23] on Petri plates containing starch nitrate agar (SNA) medium (g/l): starch 20, KNO_3_ 2, K_2_HPO_4_ 1, MgSO_4_·7H_2_O 0.5, NaCl 0.5, CaCO_3_ 2, FeSO_4_·7H_2_O 0.01, agar 20, and distilled water up to 1 L, pH 7.0 ± 0.2. The plates were incubated at 30°C for 7 days.

### Screening for Antibacterial Activity

Fresh seed cultures of the isolated actinomycetes were prepared by inoculating three cork borer disks of 9-mm diameter taken from a 7-day-old culture in a 250 ml conical flask containing 100 ml of starch nitrate broth (SNB) medium (pH 7.2 ± 0.2). Then, the inoculated flasks were incubated in a rotary incubator shaker at 150 rpm and 30°C for 48 h. To obtain CFFs, the cultures were filtered using a Whatman filter (0.45 μm) and then subjected to centrifugation at 10,000 ×*g* for 15 min. The obtained CFFs were screened for their antibacterial activity on MHA using the agar-well diffusion method [24] against 24 h young cultures of 6 standard test strains and the isolated MDR-ESKAPE pathogens. Wells (6 mm) were cut using a sterile cork borer, and 100 μl of CFF of each actinomycete was transferred to each well individually. The standard commercial antibiotic amikacin (30 μg) was used as a positive control. The inoculated plates were left for 2 h at 4°C and then incubated at 37°C for 24 h. After incubation, the inhibition zone (IZ) was measured (mm) and recorded as recommended by the National Committee for Clinical Laboratory Standards [25].

### Identification of the Most Potent Isolate, BOGE18 Cultural and morphological characteristics

Cultural characteristics including color of aerial mass, substrate mycelium, and diffusible pigments, were recorded for the isolate BOGE18 by growth on International *Streptomyces* Project (ISP 1-7) media at 30°C for 7–14 days, and the color series were recorded as described by Shirling and Gottlieb [26]. The morphology of spore-bearing hyphae with the entire spore chain was investigated under a light microscope (400×) using the coverslip culture technique, in which the culture was transferred to the base of cover slips interred in SNA medium and incubated at 30°C for 7 days. The spore surface was examined at different magnifications with a scanning electron microscope (SEM) according to the method of Tresner *et al*. [27]. The morphological features of the spore chain and spore surface were then studied according to Bergey’s Manual of Systematic Bacteriology [28].

### Chemotaxonomic Analyses

The isolate BOGE18 was allowed to grow on SNA medium at 30°C for 7 days, and the cells were scraped from the plates and then collected for analysis of the chemical composition. The isomer of diaminopimelic acid (LL-DAP or meso-DAP) was determined with paper chromatography of the hydrolyzed cells in 6 N HCl based on the method described by Becker *et al*. [29]. The whole cell sugar pattern was determined with thin-layer chromatography of hydrolyzed cells in 1 N H_2_SO_4_ according to the method of Lechevalier and Lechevalier [30].

### Physiological and Biochemical Characteristics

The ability of the isolate to utilize different carbon/nitrogen sources was determined by growth on basal medium supplemented with a 1% carbon/nitrogen source at 30°C. Other physiological characteristics were determined on ISP-2 medium by growing at different pH values (4–10), temperatures (10–60°C), NaCl concentrations (1-9%) and growth inhibitors (phenol 0.1%, sodium azide 0.01% and crystal violet 0.001%). Additionally, the biochemical tests included lipid, starch, gelatin and casein hydrolysis tests; degradation ability of tyrosine, urea, pectin, esculin and lecithin; citrate utilization; and H_2_S production were also examined. All experiments were performed at 30°C and the results were recorded after 7 days of incubation. The physiological and biochemical characteristics were tested according to the established methods described by Williams *et al*. [31].

### 16S rRNA Sequencing and Phylogenetic Analyses

The isolate was allowed to grow on ISP-2 liquid medium at 30°C for 3 days, fresh biomass (50 mg) was collected, and genomic DNA was extracted according to the method of Miller *et al*. [32]. The 16S rRNA gene was amplified with PCR using a genomic DNA template and two universal primers: 27f (5-AGAGTTTGATCCTGGCTCAG-3) and 1492r (5-GGTTACCTTGTTACGACTT-3) [33]. PCR was performed in a Thermal Cycler with a 3 min hot starting performed at 94°C, 30 cycles at 94°C for 30 s, 55°C for 30 s, and 72°C for 1 min, followed by gene extension for 10 min at 72°C. Automated sequencing was performed following previously published procedures [34].

The obtained 16S rRNA gene sequence was compared with the reference sequences available on the National Center for Biotechnology Information (NCBI) GenBank database using the Basic Local Alignment Search Tool (BLAST). Sequence alignment was performed by the CLUSTAL W program. The phylogenetic tree was inferred using the maximum likelihood method and the Tamura 3-parameter model with bootstrap testing (1,000 replicates) and application of the maximum parsimony method in MEGA11 [35].

### Cultivation and Extraction of Active Metabolites

A 1-L flask containing 250 mL of the production (SNB) medium was inoculated with (10%, v/v) fresh seed culture of the isolate BOGE18 and incubated for 7 days at 30°C with shaking at 150 rpm. After fermentation, the culture broth was filtered using a Whatman filter (0.45 μm) and finally centrifuged at 10,000 ×*g* for 30 min at 4°C. The CFF containing active metabolites was extracted twice by ethyl acetate (1:1 v/v) in a separatory funnel. After mixing and shaking, the ethyl acetate layer was collected and concentrated by evaporation under reduced pressure at 45°C in a rotary evaporator. The obtained residue was dried and stored at -20°C to evaluate its biological activities [36].

### Evaluation of the Biological Activities of BOGE18 Extract Determination of MIC

MIC was determined using a resazurin broth microdilution assay against the same (standard and MDR-ESKAPE) bacterial strains according to the method of Castilho *et al*. [37] with a slight modification. Briefly, 190 μl of MHB medium and 10 μl of stock solutions (625, 1250, 2500, 5000, 10000, 20000 μg/ml) of BOGE18 extract were mixed into the wells of a 96-well plate to obtain a final concentration (31.25, 62.5, 125, 250, 500, 1000 μg/ml) of the extract. Fifty microliters of bacterial suspension (1 × 10^5^ CFU/ml) prepared in MHB medium was inoculated into each well, and then the plates were incubated at 37°C for 24 h. The MIC values were visually determined by the color change of resazurin dye. For determination of minimum bactericidal concentration (MBC), an aliquot of 1 × MIC, 2 × MIC, and 4 × MIC cultures were plated on MHA and incubated at 37°C for 24 h. The lowest concentration that yielded no growth after this subculturing was taken as the MBC.

### In Vitro Anti-Biofilm Ability

The anti-biofilm ability was evaluated using 96-well microtiter (flat bottom, polystyrene) plates according to the method of Kalishwaralal *et al*. [38] with some modifications. Briefly, individual wells of sterile microtiter plates were filled with 180 μl of MHB and inoculated with 10 μl of overnight bacterial culture suspension (OD_620_ 0.05 ± 0.02). To the mixture, 10 μl of the extract at concentrations of ½ × MIC, ¼ × MIC, 1/8 × MIC, and 1/16 × MIC were added, and then the plates were incubated for 24 h at 37°C. Biofilms formed were fixed with absolute alcohol, flooded with crystal violet stain (0.1%, w/v) and incubated for 30 min. After drying, 200 ml of 33% acetic acid was added, and the optical densities (OD) of stained adherent bacteria were determined with a microplate reader at 630 nm. The percentage of inhibition of biofilm formation was calculated using the following formula:

$$\text{Biofilm inhibition (\%)}=1-\frac{{\text{OD}}_{\text{630 }}\text{of cells treated with different concentration of BOGE18 extract}}{{\text{OD}}_{\text{630}}\text{ of non treated control}}\times 100.$$

### CLSM Analysis of Biofilm Structure

Analysis of biofilm structure was performed according to the method of Singh *et al*. [39]. Briefly, two biofilms of MDR-ESKAPE pathogens, *S. aureus* WS12 and *A. baumannii* SC6, were prepared and treated as described in the above section. The nontreated (wells without extract as a positive control) and treated (wells with ½ × MIC and ¼ × MIC) biofilms as well as the negative control (well containing MHB media without test organism) were incubated for 48 h at 37°C. The plates were gently rinsed with saline and stained using a LIVE/DEAD BacLight Bacterial Viability Kit (Invitrogen Molecular Probes, USA). The morphology of biofilms formed was examined via CLSM (LSM 980: ZEN 3.3, Carl Zeiss, Germany) at the Cancer Children’s Hospital, Cairo, Egypt.

### Antioxidant Assays

**DPPH radical scavenging assay**

The DPPH assay was performed according to the method of Attimarad *et al*. [40]. A methanol DPPH solution (0.15%) was mixed with different concentrations (7.81-1000 μg/ml) of extract, and after 10 min, the absorbance was read at 515 nm. The antiradical activity was expressed as 50% inhibition (IC_50_ μg/ml). Vitamin C (ascorbic acid) was used as a standard. The scavenging activity was calculated using the following formula:

$$\text{DPPH scavenging activity (\%)}=\frac{\text{Absorbance (control) -Absorbance (BOGE18 extract)}}{\text{Absorbance (control)}}\times 100.$$

**ABTS radical scavenging assay**

The ABTS assay was carried out according to the method described by Siddhuraju and Manian [41] with slight modification. Briefly, ABTS radical solution was mixed with different concentrations (7.81-1000 μg/ml) of extract, and after 20 min of incubation in the dark at room temperature, the absorbance was read at 734 nm. The antiradical activity was expressed as IC_50_ (μg/ml) using vitamin C (ascorbic acid) as a standard. The percentage of ABTS scavenging activity was calculated using the formula:

$$\text{ABTS scavenging activity (\%)}=\frac{\text{Absorbance (control)-Absorbance (BOGE18 extract)}}{\text{Absorbance (control)}}\times 100.$$

### Cytotoxic Activity

**Cell culture cultivation.** Two human cancer cell lines, MCF-7 and HePG2, as well as two normal cell lines, human lung fibroblast (Wi-38) and African green monkey kidney (VERO), were obtained from Nawah Scientific Inc. (Mokatam, Egypt) and used for the cytotoxicity assay. The tested cells were maintained in Dulbeccós Modified Eagle Medium supplemented with 100 mg/ml streptomycin, 100 units/ml penicillin, and 10% heat-inactivated fetal bovine serum in a humidified, 5% (v/v) CO_2_ atmosphere at 37°C. The percentage of cell viability was calculated using the following formula:

$$\text{Cell viability (\%)}=\frac{\text{OD of treated cells}}{\text{OD control}}\times 100.$$

### MTT Cytotoxicity Assay

Aliquots of 50 μl cell suspension (3 × 10^3^ cells) were seeded in 96-well microplates and incubated in complete media for 24 h. Then, cells were treated for 48 h with another aliquot of 50 μl media containing the extract (dissolved in 0.5 % DMSO) at different concentrations (200-6.25 μg/ml). The plates were incubated at 37°C and 5% CO_2_ atmospheric conditions for 24 h. The cells were incubated with 50 μl/well of (3-(4,5-dimethylthiazol-2-yl)-2,5-diphenyltetrazolium bromide (MTT) solution. The absorbance of each well was read at a wavelength of 560 nm using an ELISA reader [42]. The negative control (DMSO, 0.5%), and positive control (curcumin, 25-3.12 μg/ml) were included. Both Wi-38 and VERO cells were used as a normal cell model for determination of the selective index (SI), which was calculated by dividing the IC_50_ of the cancer cell by the IC_50_ of the normal cell line. The morphological changes in cancer and normal cells were investigated after incubation for 24 h with the tested concentrations using an inverted phase contrast microscope.

### GC–MS Analysis of BOGE18 Extract

The chemical components of the extract were identified by GC–MS analysis according to the method of Zothanpuia *et al*. [43] with minor modifications. Briefly, the extract was dissolved in methanol, dehydrated with anhydrous sodium sulfate, and then filtered through a syringe filter (0.45 μm pore size) before injection. A mass spectrometer (Trace GC Ultra-ISQ, Thermo Scientific, USA) was used for chromatographic analysis. The column temperature was initially 70°C and then increased by 5°C/min to 280°C with hold for 2 min and then increased to 300°C at 10°C/min. The extract components were determined and identified with the mass spectra and retention time database of the Wiley 09 and NIST 11 libraries.

### Statistical Analysis

All experiments were performed in triplicate, and the data are expressed as the mean ± SD, which was calculated by using Minitab 18 software extended with a statistical package and Microsoft Excel 365. A difference was considered statistically significant when *p* ≤ 0.05.

## Results

### Isolation and Identification of MDR Bacteria

During our screening for MDR bacterial species from the collected clinical specimens, 160 clinical pathogens were isolated using different cultivation media. The distribution of these pathogens among the various clinical specimen categories varied (Table S1). In summary, the most pathogens were identified from abscess swabs (40%, *n* = 64), followed by urine cultures (25%, *n* = 40), wound swabs (12.5%, *n* = 20), throat swabs (10%, *n* = 16) and tooth swabs (7.5%, *n* = 12), while sputum (5%, *n* = 8) contained the fewest pathogens. Additionally, the number of gram-negative isolates (60%, *n* = 96) was greater than that of gram-positive isolates (40%, *n* = 64).

AST of the isolated pathogens was performed using automated VITEK 2 AES analysis. In this system, 15 antibiotics (belonging to 10 classes) and 16 antibiotics (belonging to 8 classes) were used to test the susceptibility of gram-positive and gram-negative species, respectively. The results showed that among the tested pathogens, six species coded UC11 (resistant to 16 antibiotics of 8 classes), SC6 (resistant to 15 antibiotics of 7 classes), UC22 (resistant to 9 antibiotics of 6 classes), UC36 (resistant to 3 antibiotics of 3 classes), WS12 (resistant to 9 antibiotics of 7 classes), and TS7 (resistant to 5 antibiotics of 4 classes) were selected as MDR species (Table S2, A&B).

Identification of the selected MDR species was carried out with the automated VITEK 2 system using three reagent cards, GN (gram-negative fermenting and non-fermenting bacilli), GP (gram-positive cocci and non-spore-forming bacilli), and BCL (gram-positive spore-forming bacilli). The results showed that these isolates belonged to the ESKAPE group, where the identified species were *Enterococcus faecium* TS7, *Staphylococcus aureus* WS12, *Klebsiella pneumoniae* UC11, *Acinetobacter baumannii* SC6, *Pseudomonas aeruginosa* UC22 and *Enterobacter aerogenes* UC36 with identification probabilities of 87, 87, 99, 99, 99, and 99%, respectively. Based on the manufacturer's instructions, the confidence level of identification was rated excellent (for probabilities 96 –99%) for all species except *E. faecium* TS7 and *S. aureus* WS12, which was acceptable (for probabilities 85 – 88%).

### Antibacterial Activity from Actinomycetes

**Fig. 1:**
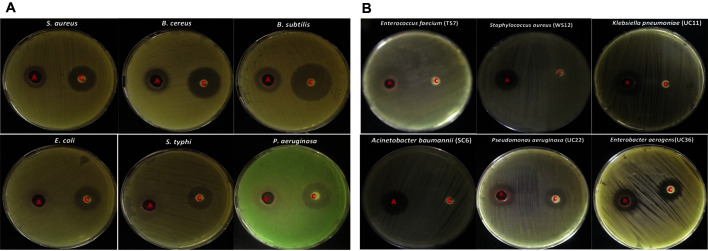
Antibacterial activity of actinomycete isolate BOGE18. (**A**) Standard test strains. (**B**) MDR-ESKAPE pathogens. In each plate, A is actinomycete (CFF), and c is control (amikacin 30 μg).

A total of 23 actinomycete isolates were screened for their antagonistic activity against both standard and MDR-ESKAPE bacterial strains. Only six isolates (26.0%) exhibited antibacterial activity against the tested strains with varying degrees, and isolate BOGE18 was the most promising due to its ability to exhibit a broad antibacterial spectrum against the tested strains (Fig. 1).

For the standard test strains, the highest inhibition activity was recorded against gram-positive organisms, where the IZ diameters ranged from 18.5 ± 0.28 to 20.6 ± 0.33 mm compared to the IZ diameters (15.6 ± 0.33 - 20.3± 0.33 mm) of gram-negative organisms. For MDR-ESKAPE pathogens, the inhibition was also higher in gram-positive species (IZ diameters ranged from 18.3 ± 0.88 to 27.66 ± 0.33 mm) than in gram-negative species (16.66 ± 0.33 - 20.33 ± 0.33 mm). Among all tested organisms, MDR *E. faecium* TS7 and *Bacillus subtilis* ATCC 6633 were the most sensitive, with IZ diameters of 27.66 ± 0.33 and 20.6 ± 0.33 mm, respectively. In addition, *E. coli* ATCC 8739 and MDR *E. aerogenes* UC36 were the least sensitive organisms, with IZ diameters of 15.6 ± 0.33 and 16.66 ± 0.33, respectively.

### Identification of *Streptomyces lienomycini* BOGE18

**Fig. 2:**
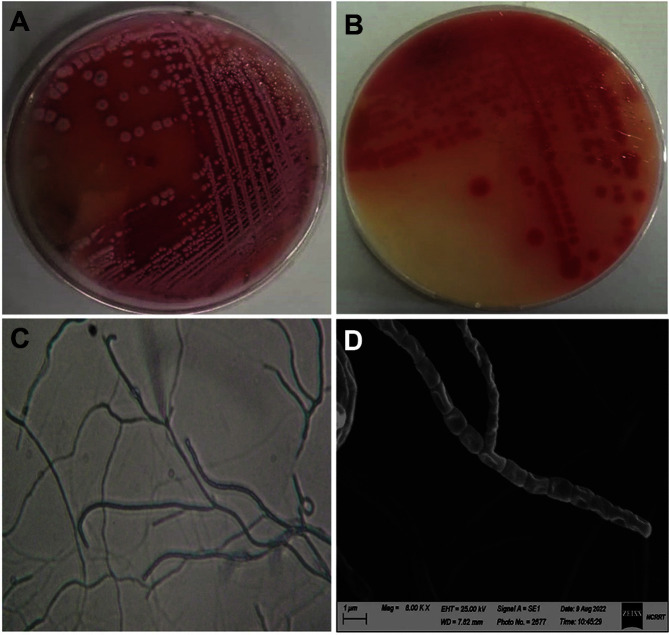
Cultural and morphological characteristics of actinomycete isolate BOGE18. (**A**) Color of aerial mycelium. (**B**) Color of substrate mycelium and diffusible pigments produced on ISP-4 medium. (**C**) Aerial hyphae bearing recti-flexible spore chains under light microscopy (400×). (**D**) SEM micrograph (8,000×) showing recti-flexible spore chains with smooth spore surfaces.

**Conventional identification**. The culture characteristics (Table S3) showed that good growth was recorded for the organism on all tested media except ISP-5 and ISP-6, on which it was weak. The mature aerial mycelium color varied from light grayish-red to strong reddish-orange, indicating that it belongs to the red‒orange color series (Fig. 2A). Deep reddish-orange diffusible pigments (Fig. 2B) are produced only on ISP-1 and ISP-4 media, while the production of melanoid pigments was not recorded. Light microscopy investigation (400×) showed that the spore chains were recti-flexible (Fig. 2C). SEM investigation (8000×) revealed recti-flexible chains of cylinder-shaped spores with a smooth surface (Fig. 2D).

The chemotaxonomic analyses showed that the organism contains glycine and LL-diaminopimelic acid (LL-DAP), suggesting that the cell wall peptidoglycan belongs to type I (Wall-chemo type I); however, no distinctive sugars were found. As a result, the isolate exhibits the same symbolic cellular component composition as the genus *Streptomyces*.

The physiological and biochemical characteristics (Table S4) revealed that the organism was able to utilize L-arabinose, L-xylose, L-rhamnose, D-glucose, D-mannitol and D-fructose as sole carbon sources for good growth. It also used the tested amino acids as the only nitrogen source, except for L-valine and L-asparagine. The highest growth was recorded at 30°C and pH 8.0, suggesting an alkali-mesophilic nature. Additionally, it was able to hydrolyze starch, lipids, gelatin, urea, and degrade tyrosine, pectin, and citrate. This information could be useful in future work to tune the medium to achieve a greater yield of bioactive compounds.

### Molecular Identification

**Fig. 3:**
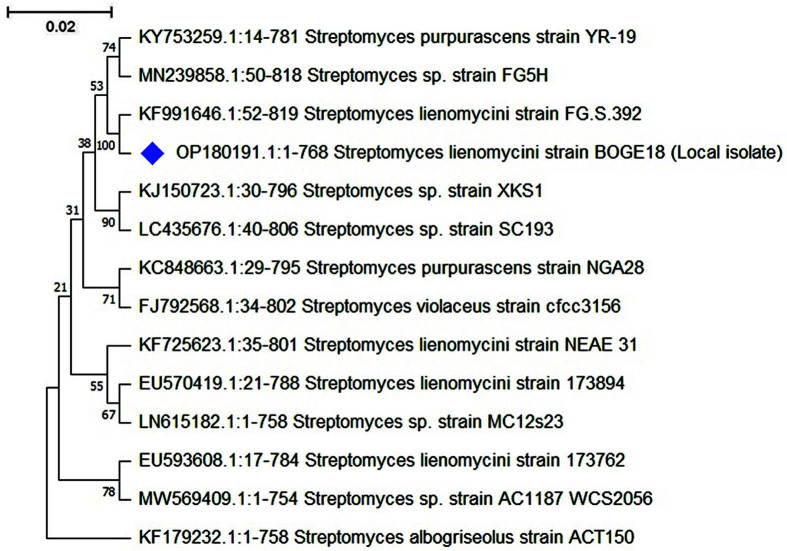
Phylogenetic tree inferred by maximum likelihood method based on 16S rRNA gene sequences using software package MEGA 11.0. The percentage of trees in which the associated taxa clustered together is shown below the branches.

The conventional identification was confirmed by molecular phylogeny of 16S rRNA sequencing. The 16S rRNA gene (768 bp) was submitted to the GenBank database (Accession No. OP180191.1). The phylogenetic tree built with the maximum likelihood method (Fig. 3) shows the relationships between isolate BOGE18 and related species of the genus *Streptomyces*. This analysis included 14 nucleotide sequences. All positions containing gaps and missing data were eliminated (complete deletion option). The results of the phylogenetic analysis revealed that the isolate belongs to a single unique subclade with *Streptomyces lienomycini* strain FG.S.392 (GenBank Accession No. KF991646.1), with which it shared 100% 16S rRNA gene sequence similarity, so it was named *S. lienomycini* BOGE18.

### Biological Capabilities of *S. lienomycini* BOGE18-Derived Extract

*S. lienomycini* BOGE18 was cultivated on a medium scale (5 L), and the fermentation broth was collected, filtered (4.6 L total volume) and extracted with ethyl acetate. The active metabolites in the BOGE18 extract were condensed using a rotary evaporator under reduced pressure, resulting in a deep, reddish-colored dry extract (2.7 g) redissolved in ethyl acetate to assess the various activities.

### MIC and MBC Determination

The MIC of the BOGE18 extract was determined for the standard and MDR-ESAPE pathogens using a broth microdilution assay (Fig. S1) and recorded (Table 1). The values varied between 62.5 and 250 μg/ml; the lowest MIC value (62.5 μg/ml) was recorded with *B. subtilis* ATCC 6633 and both MDR *P. aeruginosa* UC22 and *K. pneumoniae* UC11. The highest MIC (250 μg/ml) was recorded with *P. aeruginosa* ATCC 9072, *E. coli* ATCC 8739, and MDR *S. aureus* WS12. Concerning MBC, the highest value was recorded at 1000 μg/ml for *P. aeruginosa* ATCC 9072 and MDR *S. aureus* WS12, while the lowest MBC was at 125 μg/ml for both MDR *P. aeruginosa* UC22 and *K. pneumoniae* UC11. Additionally, moderate MBC values (250-500 μg/ml) were recorded for the other bacterial species, as shown in Table 1.

### Anti-Biofilm Ability

**Fig. 4:**
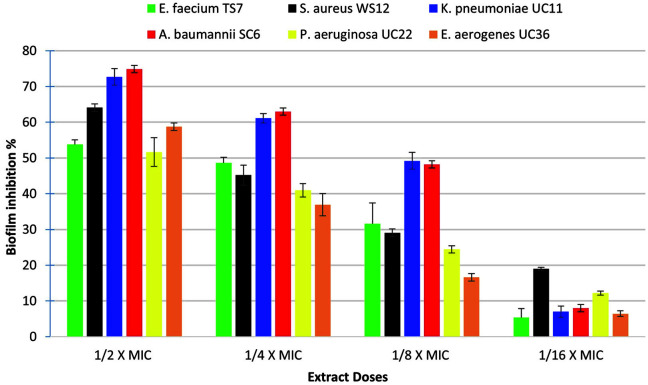
Dose-dependent inhibition of *S. lienomycini* BOGE18 extract on biofilm formation of MDR-ESKAPE pathogens. The values represent the mean ± SD of triplicate experiments (*n* = 3, *p* < 0.05).

The results of the in vitro anti-biofilm ability of the BOGE18 extract against MDR-ESKAPE pathogens (Fig. 4) demonstrated that the extract significantly (*p* < 0.05) reduced biofilm formation in a concentration-dependent manner for all tested species. The highest inhibition was recorded with *A. baumannii* SC6, where the reduction ranged from 74.87 ± 2.80% to 48.17 ± 3.84% at concentrations of 62.5 and 15.62 μg/mL, respectively. In contrast, the lowest reduction was recorded with *P. aeruginosa* UC22, with the percent of inhibition ranging between 51.63 and 12.181% at concentrations of 31.25 and 7.8 μg/mL, respectively.

### CLSM Analysis

**Fig. 5:**
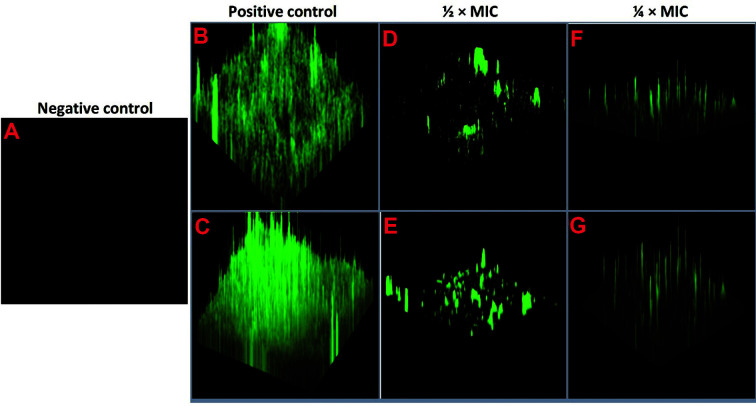
CLSM analyses of biofilm inhibition by *S. lienomycini* BOGE18 extract. (**A**) Negative control (a glass coverslip loaded with medium without the test organism). (**B**) *S. aureus* WS12. (**C**) *A. baumannii* SC6. Both are biofilms formed in the absence of the extract treatment (positive control). (**D**) *S. aureus* WS12. (**E**) *A. baumannii* SC6. Both are biofilms formed upon treatment with ½ × MIC of the extract. (**F**) *S. aureus* WS12. (**G**) *A. baumannii* SC6. Both are biofilms formed in the presence of ¼ × MIC of extract.

A double-staining CLSM technique was used to evaluate the effect of extract BOGE18 on the morphology of biofilms created by *S. aureus* WS12 and *A. baumannii* SC6 on coverslips (Fig. 5). The evaluation revealed that when sub-MIC concentrations (½ × MIC and ¼ × MIC) of extract were added during bacterial growth, the extract attenuated biofilm formation in a dose-dependent manner. The negative control (Fig. 5A) was a medium without the test organism. Untreated cells of *S. aureus* WS12 (Fig. 5B) and *A. baumannii* SC6 (Fig. 5C) are the positive controls of biofilm formation and were stained green, indicating high viability and uninhibited biofilm formation, where they exhibited a compact and condensed biofilm matrix without damage. The CLSM images acquired in the presence of ½ × MIC of the extract for *S. aureus* WS12 (Fig. 5D) and *A. baumannii* SC6 (Fig. 5E) showed a large attenuation in biofilm formation, where they exhibited a broken and light biofilm matrix when compared to untreated (positive control) cells. In contrast, CLSM images acquired in the presence of ¼ × MIC of *S. aureus* WS12 (Fig. 5F) and *A. baumannii* SC6 (Fig. 5G) showed a relatively large proportion of biofilms formed by the two bacterial species, indicating the low activity of the extract at this concentration. The results of CLSM analysis confirmed the results obtained from the quantitative assay.

### Antioxidant Capabilities

**Fig. 6:**
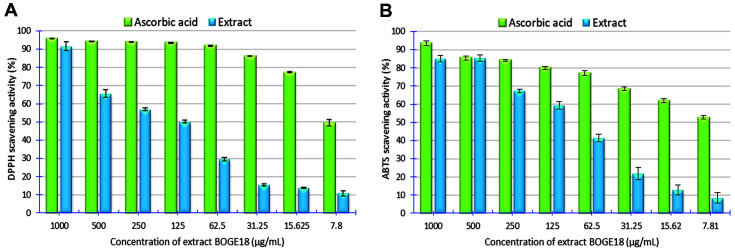
Antioxidant activities demonstrated by *S. lienomycini* BOGE18 extract using two superoxide radical scavenging assays. (**A**) DPPH. (**B**) ABTS. The values represent the mean ± SD of triplicate experiments (*n* = 3, *p* < 0.05).

The extract BOGE18 exhibited a significant dose-dependent scavenging activity (*p* < 0.05) ranging from 10.82± 2.5% to 91.61 ± 4.1% DPPH radical reduction at concentrations between 7.8 and 1000 μg/ml, with an IC_50_ of 125 μg/ml compared with the IC_50_ of vitamin C, which was 8 μg/ml (Fig. 6A). Another antioxidant technique utilized to investigate the radical scavenging activity of the extract BOGE18 was the ABTS assay, and the results demonstrated considerable ABTS radical scavenging activity (*p* < 0.05) varying from 8.49 ± 4.92% to 85.06 ± 3.14% at concentrations between 7.8 and 1000 μg/ml, with IC_50_ values of 95.3 and 7.2 μg/ml for ABTS and standard, respectively (Fig. 6B).

### Cytotoxic Activity

**Fig. 7:**
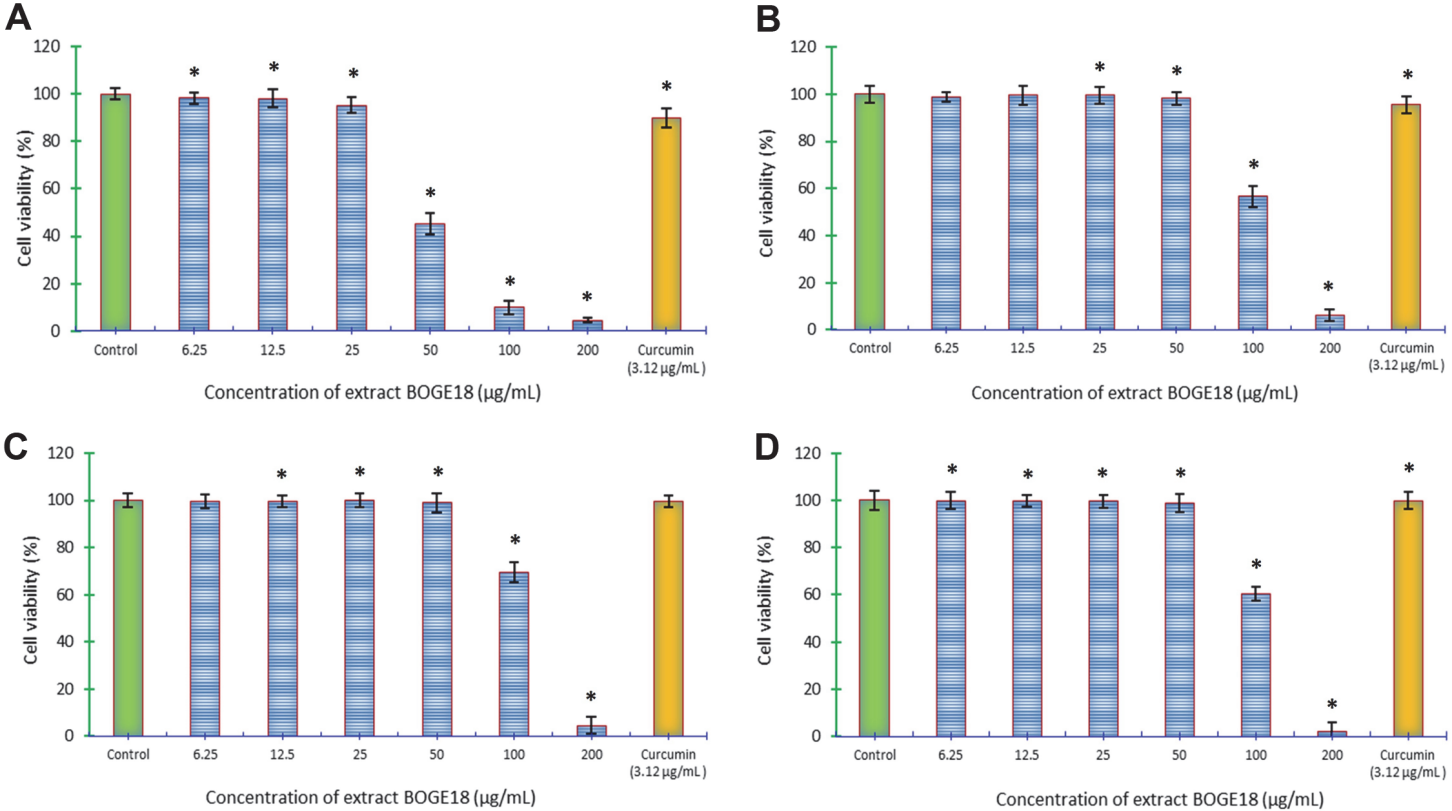
Cytotoxic effect of *S. lienomycini* BOGE18 extract on cancer and normal cell lines. (**A**) MCF-7. (**B**) HePG2. Both human cancer cell lines were used to study anticancer activity. (**C**) Wi-38. (**D**) VERO. Both are normal cells used for determination of SI. *Indicates a significant difference (*p* < 0.05) compared with the control (DMSO).

The extract BOGE18 showed varying degrees of inhibitory capacity in cancer cell growth, where MCF-7 exhibited the highest susceptibility to the extract (Fig. 7A), with the lowest IC_50_ (47.15 ± 13.10 μg/ml), while HepG2 was less sensitive with an IC_50_ of 122.69 ± 9.12 μg/ml (Fig. 7B). Concerning the cytotoxicity of the extract toward the tested normal cell lines, it was found to be less cytotoxic to Wi-38 cells with an IC_50_ of 128.86 ± 13.2 μg/ml (Fig. 7C) and VERO cells with an IC_50_ of 122.73 ± 17.10 μg/ml (Fig. 7D). Also, the SI value of the extract was recorded at 0.376 for MCF-7 and 0.975 for HepG2 cancer cell lines.

Additionally, our findings showed that the tested concentrations of extract BOGE18 caused morphological alterations in the cancerous cells, including the destruction of cell sheets, cell shrinkage, irregular cell shape, and cytoplasmic condensation, compared with the untreated cell lines (Fig. S2A). Regarding the morphology of the normal cells, they showed some changes only when treated with the highest concentration (200 μg/ml), while at low concentrations (12.5 and 6.25 μg/ml), they maintained their usual morphological appearance (Fig. S2B).

### GC–MS Analysis of *S. lienomycini* BOGE18-Derived Extract

The GC‒MS analysis of the BOGE18 extract (Fig. S3) revealed nine recognized compounds in its constituents. The identified compounds (Fig. S4) are hexadecanoic acid, methyl ester (**1**); hexadecanoic acid (**2**); 9,12-octadecadienoic acid (Z, Z)-, methyl ester (**3**); 9-octadecenoic acid, methyl ester, (E)- (**4**); octadecanoic acid, methyl ester (**5**); 9-octadecenoic acid (oleic acid) (**6**); 13-docosenoic acid, methyl ester, (Z)- (**7**); dodecanoic acid, 1,2,3-propanetriyl ester (**8**) and octadecanoic acid, 2-[(1-xododecyl) oxy]-1,3-propanediyl ester (**9**). All substances found were identified, and every compound peak area was proportionate to its concentration in the extract. Comparing the peaks' mass spectra to those in the NIST database aided in identifying them by relying on molecular formula, retention time, and molecular mass (Table 2).

Aliphatic acids (fatty acids) were identified as the predominant category, among which 1,2,3-propanetriyl ester (35.72%); dodecanoic acid, octadecanoic acid, 2-[(1-xododecyl) oxy]-1,3-propanediyl ester (20.11%) and 9-octadecenoic acid, methyl ester, (E)- (10.47%) were found to be the main constituents. Hexadecanoic acid methyl ester, hexadecanoic acid (palmitic acid), 9,12-octadecadienoic acid (Z, Z)-, methyl ester, octadecanoic acid, methyl ester, 9,12-octadecadienoic acid (Z, Z)- and 13-docosenoic acid, methyl ester,(Z)- were minor components, and their peak area ratios ranged from 1.03 to 5.01%. Also, 9-octadecenoic acid (oleic acid) (0.77%) and cis-11-eicosenoic acid (0.14%) were considered trace compounds.

## Discussion

Currently, there are many cases of MDR bacteria worldwide, which is a significant public health problem. During our screening program for the isolation and identification of MDR bacteria, a total of 160 clinical pathogens were recovered from various clinical specimens collected from some Egyptian hospital patients. Gram-negative species (60%, *n* = 96) were more prevalent than gram-positive species (40%, *n* = 64). These findings were consistent with those of other researchers studying the incidence of nosocomial pathogens in Egypt [44,45], as well as from countries other than Egypt [46].

AST of the isolated pathogens was assessed using VITEK 2 AES analysis. Compared to routine antibiotic susceptibility testing methods, VITEK 2 enables accurate performance of susceptibility testing, while the AES appropriately detects and interprets resistance mechanisms [21]. The pattern of antibiotic resistance among these pathogens varied, ranging from resistance to three classes of antibiotics to eight antibiotic classes. Among the 160 tested pathogens, six were selected as MDR species. According to Magiorakos *et al*. [47], an MDR species is resistant to at least one antibiotic in three or more antibiotic classes.

Identification of these species was carried out with the VITEK 2 automated system, which revealed these isolates as belonging to the ESKAPE group, with identification probabilities ranging from 87 to 99%. Decarli *et al*.[48] reported that the use of automated systems in the identification of clinical species can improve antibiotic therapy and significantly impact clinical outcomes by reducing antibiotic resistance, hospitalization, costs, and mortality.

According to Rice [49], MDR-ESKAPE pathogens represent an indigenous group of nosocomial organisms. One of the unexpected findings of this research was that the obtained MDR-ESKAPE pathogens represented incidence, especially their pattern of resistance to most antibiotics tested. As a result, developing an antimicrobial resistance monitoring mechanism in Egypt and implementing comprehensive recommendations for antibiotic usage is critical.

Researchers worldwide are searching intensively for healthy, bioactive, novel, and broad-spectrum postbiotic metabolites from diverse microbial sources [8, 50, 51]. Actinomycetes are prolific makers of postbiotic metabolites in natural soil habitats [52]. In this context, the soil-derived actinomycete isolate BOGE18 exhibited a promising and broad antibacterial spectrum against both the standard and MDR-ESKAPE (gram-positive and gram-negative) species, which is a promising outcome. Our findings of antibacterial activity showed that the IZ diameters ranged from 18.5 ± 0.28 to 20.6 ± 0.33 and 18.3 ± 0.88 to 27.66 ± 0.33 mm for the standard and MDR-ESKAPE gram-positive species, respectively. Meanwhile, in gram-negative species, they ranged from 15.6 ± 0.33 to 20.3 ± 0.33 and 16.66 ± 0.33 to 20.33 ± 0.33 mm for the standard and MDR-ESKAPE groups, respectively. These antibacterial findings were consistent with those obtained in a previous investigation whereby actinomycetes were isolated from soils and exhibited considerable antibacterial activity against gram-positive and negative pathogens, with IZ diameters ranging from 13 to 27 mm [53]; however, they were in contrast with those of Singh *et al*. [54], who reported the activity of soil-isolated actinomycetes against only gram-positive pathogens.

According to Abdel-Haliem *et al*. [55], the physical and chemical characteristics of Egyptian soil, as well as the soil microbial taxa, differed depending on the location and environment. El-Bahariya Oasis is a significant location in the western desert of Egypt. It is characterized by special conditions such as high alkalinity and salinity, arid hot summers, cold winters, and rare rainfall. Wilson and Brimble [56] reported that microbes must first adapt and develop to withstand diverse stress circumstances and, as a result, have the potential to create novel biochemical agents to perform specific biofunctions and bioactivities. Therefore, our isolates of actinomycetes from El-Bahariya Oasis soil represent an unexplored source for discovering valuable biologically active compounds. To our knowledge, a few reports have recorded antibacterial activity from actinomycetes against MDR-ESKAPE pathogens [57], but they have not been reported previously in Egypt.

In this study, the most promising strain, BOGE18, was identified with conventional and molecular approaches. The outcomes of cultural, morphological, physiological, and biochemical tests, together with the chemotaxonomic features of this isolate, were similar to those of the genus *Streptomyces*, based on Williams *et al*. [28] and Bergey's Manual [58, 59]. The strain shares 100% of its 16S rRNA gene sequence similarity with *S. lienomycini* strain FG.S.392. As a result of the polyphasic approaches utilized in characterization, this isolate was named *S. lienomycini* strain BOGE18. Similarly, Lim *et al*. [60] documented screening of soil-isolated actinomycetes against MDR bacteria, which were later identified as *Streptomyces* spp. based on 16S rRNA analysis, as well as the cultural and morphological characteristics and biochemical profiles.

For evaluation of the biological capabilities of the extract derived from *S. lienomycini* BOGE18, the bioactive metabolites were extracted from CFF by the organic solvent ethyl acetate. Sharma and Manhas [61] reported that ethyl acetate solvent is suitable for recovering active metabolites with different activities in a fermentation broth.

The lowest MIC value (62.5 μg/ml) of the *S. lienomycini* BOGE18 extract was lower than that reported in an earlier study by Singhania *et al*. [62], which recorded the MIC of the crude extract belonging to *Streptomyces laurentii* VITMPS at 100 μg/ml. Meanwhile, the highest MIC (250 μg/ml) was higher than that recorded by Khieu *et al*. [63]. According to Enright [64], various aspects influence the MIC value, notably test organism susceptibility, medium composition, incubation temperature, microbe type, and active metabolite concentration. Concerning the MBC, the values ranged between 125 and 1000 μg/ml. It is commonly assumed that a drug is bactericidal whenever the MBC equals or is four times less than the MIC [65]. Therefore, we report that the *S. lienomycini* BOGE18-derived extract has bactericidal characteristics.

The biofilm matrix is critical in antibiotic resistance development because it protects bacteria from the host immunity and prevents the penetration of anti-microbial drugs [66]. MDR bacteria have recently attracted the interest of microbiological researchers in the discovery of anti-biofilm agents. Numerous anti-biofilm agents were previously reported from various species of *Streptomyces*, including *Streptomyces gandocaensis* DHS334 against *A. baumannii* [67], *Streptomyces* sp. against *S. aureus* (MRSA) [16], *Streptomyces* sp. SBT343 against *S. epidermidis* [68], and *Streptomyces albus* against *Vibrio anguillarum* [69]; however, they were not reported earlier against MDR-ESKAPE pathogens together.

We have reported a significant anti-biofilm ability of the extract BOGE18 against biofilms formed by MDR-ESKAPE pathogens. Our results of the in vitro anti-biofilm assay showed that at sub-MIC concentrations (½ × MIC – 1/16 × MIC), the extract was effective in inhibiting biofilms formed by the tested pathogens. These findings were similar to those of Kemung *et al*. [15], who reported that the methanolic extract of *Streptomyces* sp. MUSC showed anti-biofilm activity against methicillin-resistant *Staphylococcus aureus* at 1/8 × MIC. In our study, we also investigated the anti-biofilm effect of extract BOGE18 on the morphology of biofilm coverslips formed by *S. aureus* WS12 and *A. baumannii* SC6 using qualitative CLSM analysis. The results showed that the cells treated with extract had changed biofilm morphology, while untreated biofilms had a standard uniform shape, and a significant proportion of bacterial cells remained alive, as evidenced by the green fluorescence amount. This indicates that bacterial cell survivability is not affected by the extract since it cannot cause any interference. These findings are similar to those reported by Balasubramanian *et al*. [68].

Given the complexity of the oxidation cycle and given that the oxidation process is quite complicated and may happen through various mechanisms, conducting only one antioxidant test is inadequate to determine the overall antioxidant capability of natural product extracts [70]. Therefore, in this study, two antioxidant assays, DPPH and ABTS, were used to examine the antioxidant characteristics of extract BOGE18, which displayed significant (*p* < 0.05) scavenging activity for the two radicals.

Several prior investigations have reported antioxidant metabolites with varying levels of DPPH and/or ABTS scavenging activity from *Streptomyces* spp., such as *Streptomyces* sp. MUSC125 [15], *Streptomyces* sp. KTM18 [17], *Streptomyces parvulus* VITJS11 [71], *Streptomyces carpaticus* MK-01 [18], and *Streptomyces puniceus* RHPR9 [72].

In the present study, the extract BOGE18 showed scavenging activity ranging from 10.82 ± 2.5 to 91.61 ± 4.1%and 8.49 ± 4.92 to 85.06 ± 3.14% of DPPH and ABTS, respectively, at concentrations of 7.8 and 1000 μg/ml, respectively. From these results, we found that the scavenging activity of DPPH and ABTS radicals increased with increasing extract concentration, which is in contrast with Subramanian *et al*. [18], who reported a decrease in DPPH scavenging with increasing extract concentration. Our findings of DPPH and ABTS radical scavenging activity are promising, as they were higher than those reported in an earlier study by Tan *et al*. [73], who recorded the scavenging activity of *Streptomyces* sp. MUM292 extract for DPPH ranged from 9.09 ± 1.40% to 35.98 ± 5.39%at concentrations of 0.5 and 4 mg/ml, and for ABTS, it ranged from 10.64 ± 1.27% to 67.96 ± 2.23% at concentrations between 0.25 mg/ml and 4 mg/ml.

Cancer is one of the most serious health problems affecting people today. Recently, natural anticancer drugs of microbial origin have received much interest due to their reported health advantages [8, 50, 74]. Actinomycetes produce secondary metabolites with a variety of valuable capabilities, including anticancer activity, especially from *Streptomyces* spp., such as *Streptomyces cyaneofuscatus* M-157 [75], *Streptomyces lusitanus* SCSIO LR32 [76], *Streptomyces* sp. M-207 [19], and *Streptomyces malaysiense* [20].

In our continuing effort to evaluate the biological capabilities of the *S. lienomycini* BOGE18 extract, we recorded potent cytotoxic activity of the extract against MCF-7 and HePG2 cell lines. MCF-7 was the most susceptible and had a lower IC_50_ of 47.15 ± 13.10 compared to the IC_50_ of HePG2 of 122.69 ± 9.12 μg/ml. The marked cytotoxicity recorded against breast cancer (MCF-7) serves as a promising outcome, since this type of cancer is one of the most prevalent cancers among women worldwide, and with an estimated 2.3 million new cases annually, it is also listed as the fifth leading cause of cancer-related deaths [77]. Our cytotoxicity results agree with those reported by Ser *et al*. [78], who found that the extract derived from *Streptomyces pluripotens* MUSC137 exhibited the highest cytotoxic activity against MCF-7 cells, with the lowest IC_50_ of 61.33 ± 17.10 μg/ml. However, it was in contrast with Osama *et al*. [74], who reported that the crude extracts obtained from *Streptomyces* spp. SH8, SH10 and SH13 exhibited the highest cytotoxicity against the HepG2 cell line, with lower IC_50_ values of 1.31, 7.27, and 9.7 μg/ml, respectively.

Substantial efforts have been made to find innovative chemotherapeutic drugs with excellent specificity and efficacy. Another promising finding of the current study is that the present investigation on the specificity of extract BOGE18 showed that the extract is less toxic to the tested normal cell lines, where the IC_50_ was recorded at 128.86 ± 13.2 and 122.73 ± 17.10 μg/ml for Wi-38 and VERO cells, respectively. Effective chemotherapeutic drugs should possess high specificity and distinguish between normal and cancerous cells. Several anticancer medications lack this specificity and destroy both healthy and cancerous cells [79].

The remarkable antibacterial and anti-biofilm activities against MDR-ESKAPE pathogens, as well as the antioxidant and cytotoxic capabilities, have prompted further investigation to analyze the chemical compounds present in *S. lienomycini* BOGE18-derived extract. GC‒MS analysis revealed the presence of nine recognized compounds in the extract. GC‒MS has previously been used to perform chemical analysis for putative compound identification in microbial bioproducts following the determination of their biological actions [80].

Compound (**1**) was discovered in a *Streptomyces werraensis* MI-S.24-3 ethyl acetate extract by GC‒MS testing and displayed cytotoxic and antibacterial activities [80]. Compounds (**2, 4** & **6**) reported earlier in *Streptomyces* sp. 1S1 ethyl acetate extract accountable for this strain's antibacterial activity was also found by GC‒MS analysis [81]. Compound (**3**) was also detected in the MeOH extract of *Streptomyces alfalfae* XN-04 with plant growth-promoting and antifungal properties [82]. Furthermore, compound (**5**) was identified in GC‒MS analysis for *Streptomyces anulatus* NEAE-94 with antibiotics against MDR *S. aureus* [83]. Compound (**7**) is a bioactive fatty acid amide, and a derivative of this compound has been isolated from plants, animals, bacteria [84], and *Penicillium griseofulvum* [85], but was not previously derived from *Streptomyces* spp. Compound (**8**) was previously described as crown daisy plant secretion (*Chrysanthemum coronarium* L.) intercropped with tomatoes (*Solanum lycopersicum*) to inhibit parasitism by root-knot nematodes (*Meloidogyne incognita*) [86]. Finally, compound (**9**) was also detected in the *Streptomyces* sp. SCA3-4 crude extracts with antimicrobial activity [87]. The current research results confirmed that the multitude of biological activities of the BOGE18 extract might be due to the presence of these compounds.

The actinomycete *Streptomyces lienomycini* strain BOGE18 which was isolated from Egyptian soil and exhibited a potential antibacterial activity against MDR-ESKAPE clinical pathogens was identified by polyphasic approaches of phenotypic, genomic, and phylogenetic analyses. The ethyl acetate extract of the CFF of this strain demonstrated significant antibacterial and anti-biofilm activity against MDR-ESKAPE pathogens, as well as antioxidant and cytotoxic capabilities. Due to the absence of cell toxicity of this extract, these combined results suggest that *S. lienomycini* strain BOGE18 metabolites are promising for future use as a natural alternative strategy against bacterial resistance. Comprehensively, this study emphasizes that Egyptian soils are a valuable origin for bioactive *Streptomyces* spp. displaying a multitude of biological activities, including anti-MDR bacteria, antibiofilm, antioxidant, and cytotoxic activities.

## Supplemental Materials

Supplementary data for this paper are available on-line only at http://jmb.or.kr.

## Figures and Tables

**Table 1:** MIC and MBC of *S. lienomycini* BOGE18 extract against standard and MDR-ESKAPE bacteria.

No	Bacterial strains	Concentration (μg/ml)	

		MIC	MBC
● Standard test strains			
1	*Staphylococcus aureus* (ATCC 6538)	125	500
2	*Bacillus cereus* (ATCC 10987)	125	250
3	*Bacillus subtilis* (ATCC 6633)	62.5	250
4	*Escherichia coli* (ATCC 8739)	250	500
5	*Salmonella typhi* (ATCC 14028)	125	250
6	*Pseudomonas aeruginosa* (ATCC 9072)	250	1000
● MDR-ESKAPE pathogens			
7	*Staphylococcus aureus* WS12	250	1000
8	*Enterococcus faecium* TS7	125	250
9	*Acinetobacter baumannii* SC6	125	500
10	*Enterobacter aerogenes* UC36	125	250
11	*Pseudomonas aeruginosa* UC22	62.5	125
12	*Klebsiella pneumoniae* UC11	62.5	125

**Table 2:** GC–MS analysis of crude ethyl acetate extract of *S. lienomycini* BOGE18.

No.	Compounds	Chemical formula	Molecular weight	Retention time (min)	Peak area (%)
1	Hexadecanoic acid, methyl ester	C_17_H_34_O_2_	270	26.44	3.87
2	Hexadecanoic acid (palmitic acid)	C_16_H_32_O_2_	256	28.10	3.51
3	9,12-Octadecadienoic acid (Z,Z)-, methyl ester	C_19_H_34_O_2_	294	29.56	5.01
4	9-Octadecenoic acid, methyl ester, (E)-	C_19_H_36_O_2_	296	29.73	10.47
5	Octadecanoic acid, methyl ester	C_19_H_38_O_2_	298	30.14	1.03
6	9-octadecenoic acid (oleic acid)	C_18_H_34_O_2_	282	31.52	0.77
7	13-Docosenoic acid, methyl ester, (Z)-	C_23_H_44_O_2_	352	36.24	3.01
8	Dodecanoic acid, 1,2,3-propanetriyl ester	C_39_H_74_O_6_	638	38.26 - 46.77	35.72
9	Octadecanoic acid, 2-[(1-xododecyl) oxy]-1,3-propanediyl ester	C_51_H_98_O_6_	806	47.39 - 47.66	20.11
