# Supplementary material for: Antagonistic Potentiality of Actinomycete-Derived Extract with Anti-Biofilm, Antioxidant, and Cytotoxic Capabilities as a Natural Combating Strategy for Multidrug-Resistant ESKAPE Pathogens
Source: J Microbiol Biotechnol. 2022 Dec 23;33(1):61–74. doi: 10.4014/jmb.2211.11026 (PMC9896001; doi:10.4014/jmb.2211.11026)
Supplement: Supplementary file 1 [file jmb-33-1-61-supple.pdf]

Table S1. Distribution of the bacterial pathogens within different clinical specimens.

No.	Clinical specimens	Isolate code	No. of pathogens	Gram stain reaction	
				Gram (+) ve	Gram (-) ve
1	Urine cultures	UC	40	16	24
2	Abscess swabs	AS	64	26	38
3	Sputum cultures	SC	8	4	4
4	Wound swabs	WS	20	6	14
5	Tooth swabs	TS	12	5	7
6	Throat swabs	TS	16	7	9

Table S2(A). Antibiotic resistance pattern of the selected Gram-negative isolates.

Isolates \ Antibiotics	Ampicillin	Ampicillin/Sulbactam	Piperacillin/Tazobactam	Cefazolin	Cefoxitin	Ceftazidime	Ceftriaxone	Cefepime	Meropenem	Amikacin	Gentamicin	Tobramycin	Ciprofloxacin	Levofloxacin	Nitrofurantoin	Trimethoprim/ Sulfamethoxazole
UC11	R	R	R	R	R	R	R	R	R	R	R	R	R	R	R	R
SC6	R	R	R	R	R	R	R	R	R	R	R	R	R	R	R	S
UC22	R	R	S	R	R	S	S	S	S	S	R	R	R	R	R	S
UC36	R	R	S	R	R	S	S	S	S	S	S	S	S	S	I	S

*R=Resistant, S=Sensitive, I=Intermediate.

Table S2(B). Antibiotic resistance pattern of the selected Gram-positive isolates.

Isolates \ Antibiotics	Benzylpenicillin	Oxacillin	Gentamicin	Ciprofloxacin	Moxifloxacin	Erythromycin	Clindamycin	Linezolid	Teicoplanin	Vancomycin	Tetracycline	Tigecycline	Fusidic acid	Rifampicin	Trimethoprim/ Sulfamethoxazole
WS12	R	R	R	R	R	S	I	S	R	S	R	S	R	S	R
TS7	R	R	R	S	S	S	S	S	R	S	S	R	S	S	S

*R=Resistant, S=Sensitive, I=Intermediate.

Table S3. Cultural characteristics of actinomycete isolate BOGE18 allowed to be grown on different ISP media.

Media	Growth rate	Colony color		Diffusible pigment
		Aerial mycelium	Substrate mycelium	
ISP medium - 1 (Liquid tryptone yeast extract)	Excellent	Strong reddish orange (ISCC-NBS 35)	Moderate reddish orange (ISCC-NBS 37)	Deep reddish orange (ISCC-NBS 36)
ISP medium - 2 (Yeast extract-malt extract agar)	Excellent	Light orange yellow (ISCC-NBS 70)	Light grayish red (ISCC-NBS 18)	None
ISP medium - 3 (Oatmeal agar)	Excellent	Strong reddish orange (ISCC-NBS 35)	Light orange yellow (ISCC-NBS 70)	None
ISP medium - 4 (Inorganic salts-starch agar)	Excellent	Light grayish red (ISCC-NBS 18)	Moderate reddish orange (ISCC-NBS 37)	Deep reddish orange (ISCC-NBS 36)
ISP medium - 5 (Glycerol asparagine agar)	Weak	Reddish gray (ISCC-NBS 18)	No sporulation	None
ISP medium - 6 (Peptone yeast extract iron agar)	Weak	Reddish gray (ISCC-NBS 18)	No sporulation	None
ISP medium - 7 (Tyrosine agar)	Excellent	Light orange yellow (ISCC-NBS 70)	Deep orange (ISCC-NBS 51)	Strong yellow (ISCC-NBS 34)

Table S4. Physiological and biochemical characteristics of the isolate BOGE18.

Characters	Growth rate	Characters	Growth rate
**(1) Physiological characteristics:**			
- Utilization of carbon sources:		- Tolerance to NaCl (%):	
D-glucose	++	1	++
L-rhamnose	++	2	++
D-xylose	++	3	++
D-mannitol	++	4	++
Inositol	+	5	+
Sucrose	+	6	+
L-arabinose	+ +	7	+
Fructose	++	8	Wg
Cellulose	+	9	−
- Utilization of nitrogen sources:		- Tolerance to growth inhibitors (w/v):	
L-asparagine	−	Sodium azide (0.01%)	−
L-cysteine	+	Phenol (0.1%)	−
L-valine	−	Crystal violet (0.0001%)	+
L-threonine	++	Potassium tellurite (0.001%)	−
L-phenylalanine	+	- Sensitivity to antibiotics:	
L-methionine	++	Penicillin G (10 IU)	+
L-histidine	++	Rifampicin 50 (µg/mL)	−
L-arginine	++	Neomycin 50 (µg/mL)	−
- Growth at different temperature (°C):		**(2) Biochemical characteristics:**	
10	−	- Hydrolysis of:	
20	+	Starch	+
30	+++	Lipid	+
40	+	Gelatin	+
50	+	Casein	−
60	−	Urea	+
- Growth at different pH values:		- Degradation of:	
4	−	Tyrosine	+
5	+	Lecithin	−
6	+	Pectin	+
7	++	Esculin	−
8	+++	Citrate	+
9	Wg	Nitrate reduction	−
10	−	H_2S production	−

"–" Negative, "Wg" weak growth, "+" Moderate, "++" Good, "+++" Abundant growth.

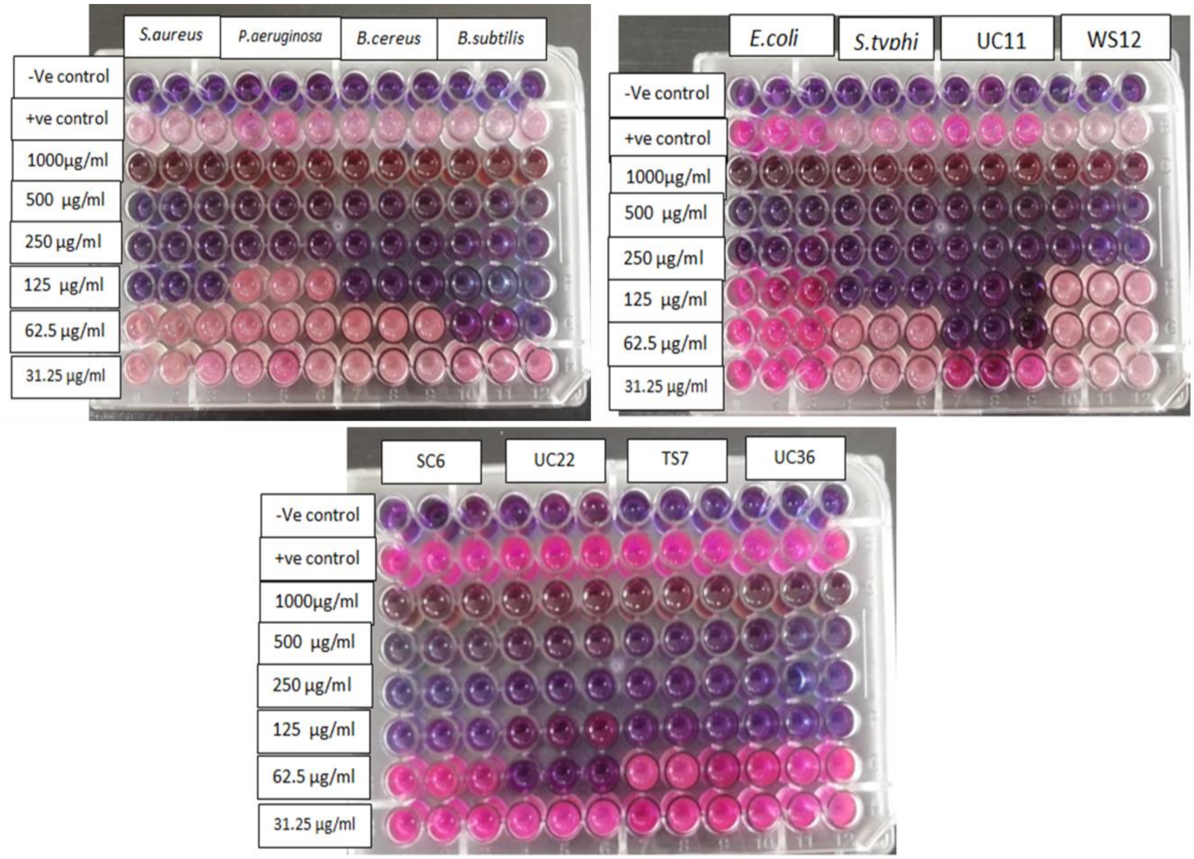

Fig. S1. Broth microdilution assay plates for MICs of *S. lienomycini* BOGE18 extract against the standard and MDR-ESAPE pathogens. These plates after 24 h in MHB resazurin assay [pink colour indicates growth and blue means inhibition of growth. 1st row (-ve) = Negative or sterility control (MHB + sterile distilled water + indicator) without bacteria; 2nd row (+ve)=Positive Control (MHB + bacterial suspension + indicator) without the extract; 3rd to 8th row (MHB + indicator + tested bacterial organism + *S. lienomycini* BOGE18 extract), the highest concentration in 3rd row then double fold dilution until the last row.

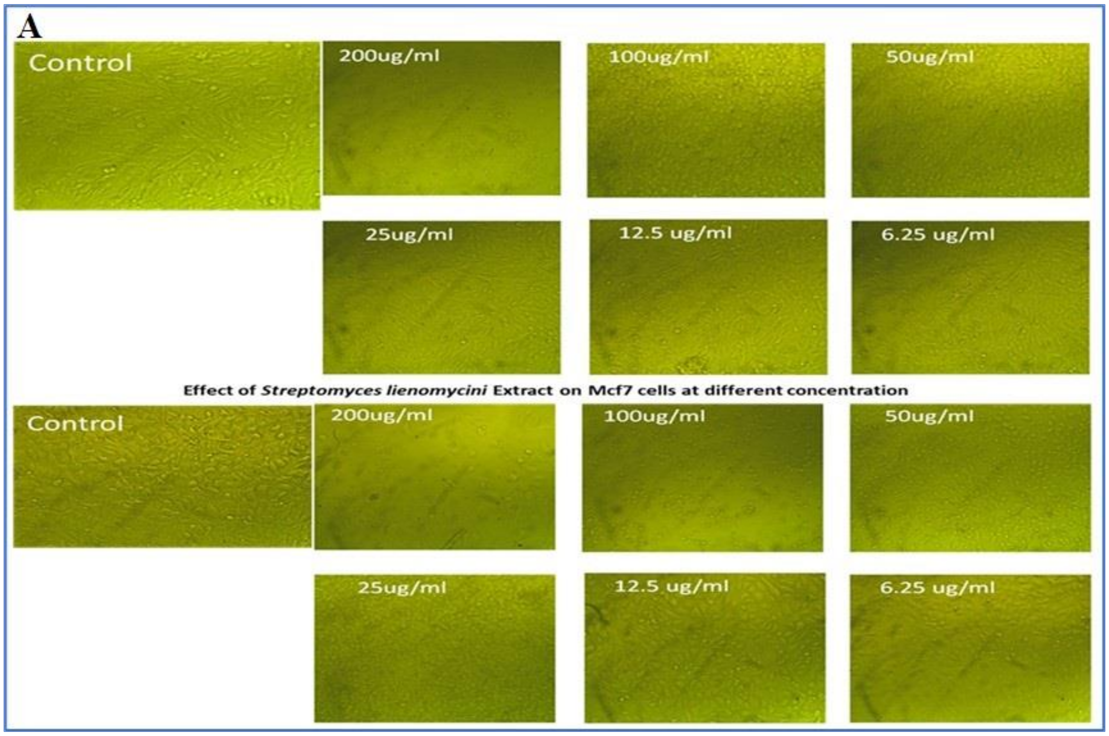

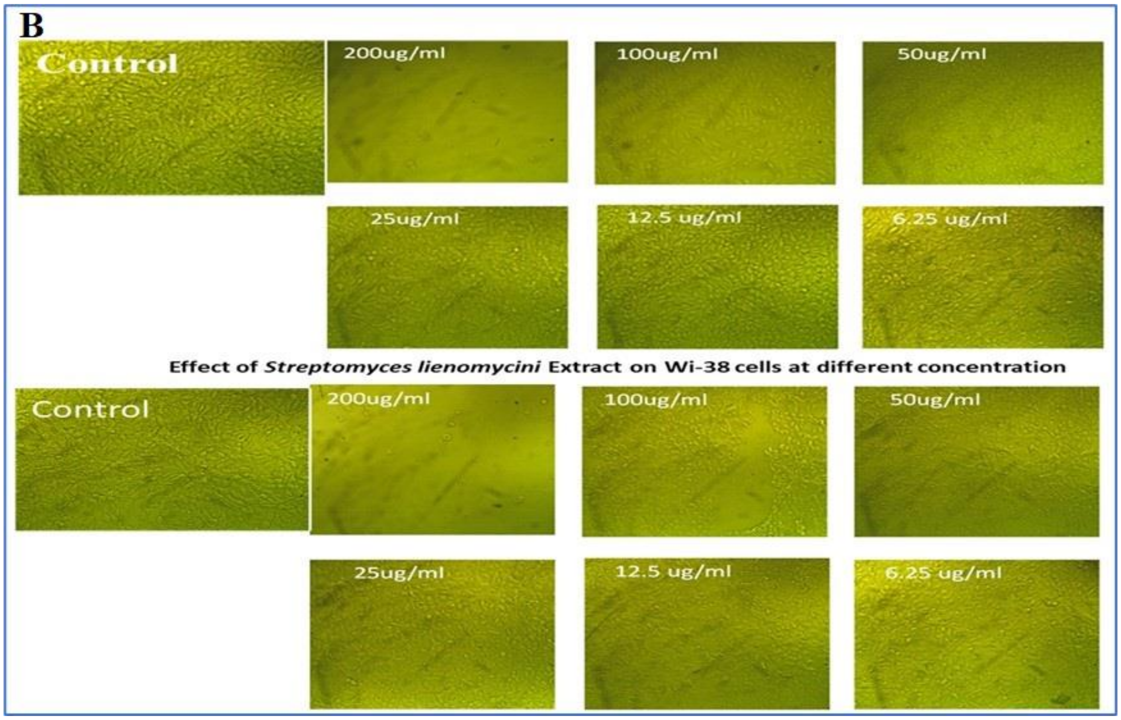

Fig. S2. Morphological changes induced by *S. lienomycini* BOGE18 extract in human cancer (MCF-7 and HePG2) cells (A), and normal (Wi-38 and VERO) cells (B).

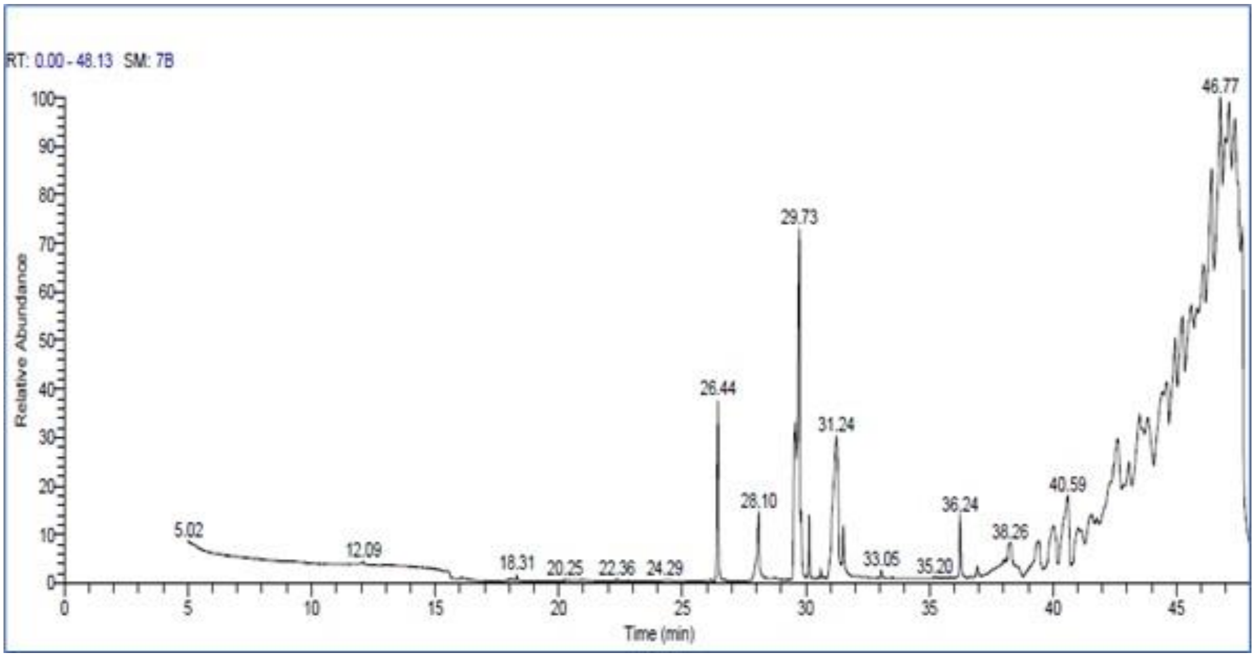

Fig. S3. GC–MS chromatogram of *S. lienomycini* BOGE18-derived extract.

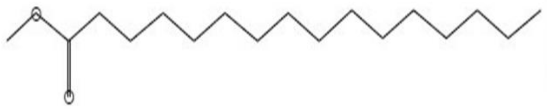

(1) Hexadecanoic acid, methyl ester

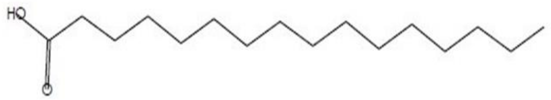

(2) Hexadecanoic acid (palmitic acid)

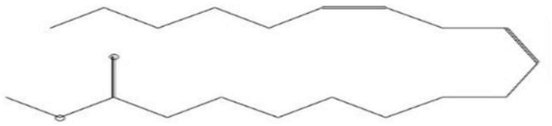

(3) 9,12-Octadecadienoic acid (Z,Z)-, methyl ester

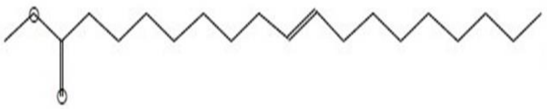

(4) 9-Octadecenoic acid, methyl ester, (E)-

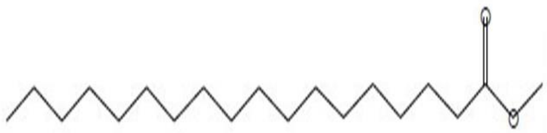

(5) Octadecanoic acid, methyl ester

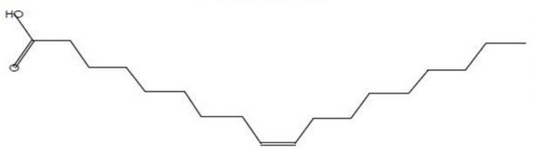

(6) 9-octadecenoic acid (oleic acid)

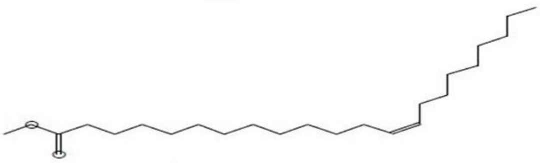

(7) 13-Docosenoic acid, methyl ester, (Z)-

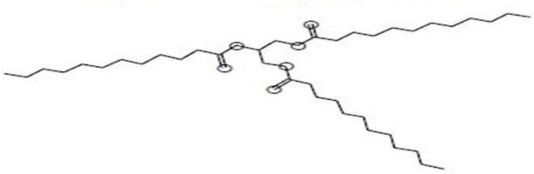

(8) Dodecanoic acid, 1,2,3-propanetriyl ester

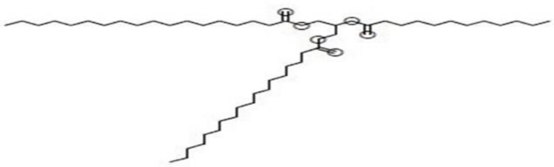

(9) Octadecanoic acid, 2-[(1-xododecyl) oxy]-1,3-propanediyl ester

Fig. S4. The molecular structures of the identified compounds from GC–MS analysis of *S. lienomycini* BOGE18-derived extract.
